# Controlled Synthesis and Visible-Light-Driven Photocatalytic Activity of BiOBr Particles for Ultrafast Degradation of Pollutants

**DOI:** 10.3390/molecules28145558

**Published:** 2023-07-20

**Authors:** Xiaohui Ji, Chen Li, Junhai Liu, Tianlei Zhang, Yue Yang, Ruijin Yu, Xuegang Luo

**Affiliations:** 1 School of Environment and Resource, Southwest University of Science and Technology, Mianyang 621010, China; snut_xiaohuiji@163.com (X.J.); leechen_317@126.com (C.L.); iamliujunhai@126.com (J.L.); ztianlei88@163.com (T.Z.); 2 Shaanxi Province Key Laboratory of Catalysis, School of Chemistry and Environmental Science, Shaanxi University of Technology, Hanzhong 723001, China; 3 College of Chemistry & Pharmacy, Northwest A&F University, Xianyang 712100, China; rrysdyx@163.com (Y.Y.); yuruijin@nwsuaf.edu.cn (R.Y.); 4 School of Life Science and Engineering, Southwest University of Science and Technology, Mianyang 621010, China

**Keywords:** photocatalysis, BiOBr, visible light, photodegradation

## Abstract

For the purpose of regulating the visible-light-driven photocatalytic properties of photocatalysts, we selected BiOBr as the research target and various routes were used. Herein, via the use of a hydrothermal method with various solvents, BiOBr particles with controllable morphology and photocatalytic activities are obtained. In particular, through changing the volume ratio of ethylene glycol (EG) to ethanol (EtOH), BiOBr compounds possess microspheres, in which samples synthesized by using EG:EtOH = 1:2 have the highest photocatalytic activity, and can completely decompose RhB under visible light irradiation within 14 min. Furthermore, we also used different volume ratios of EG and H_2_O reaction solvents to prepare BiOBr particles so as to further improve its pollutant removal ability. When the volume ratio of EG to H_2_O is 1:1, the synthesized BiOBr particles have the best photocatalytic activity, and RhB can be degraded in only 10 min upon visible light irradiation. Aside from the reaction solvent, the impact of sintering temperature on the photocatalytic properties of BiOBr particles is also explored, where its pollutant removal capacities are restrained due to the reduced specific surface area. Additionally, the visible-light-triggered photocatalytic mechanism of BiOBr particles is determined by h^+^, ·OH and ·O^2−^ active species.

## 1. Introduction

With the rapid development of modern industry, environmental pollution has received increasing attention. In particular, water pollution is becoming the focus of the world. In order to achieve wastewater purification, photocatalysis based on semiconductors has drawn considerable attention due to its amazing advantages, namely, utilizing solar energy to photodegrade pollutants or split water to produce hydrogen [1,2,3]. In recent decades, many photocatalysts, such as TiO_2_, SnO_2_, ZnO, and WO_3_, were developed to decompose the pollutants [4,5,6,7]. Nevertheless, most of them suffer from some intrinsic drawbacks including low efficiency, expensive, fast recombination of photogenerated hole–electron pairs, poor light harvest ability. As a consequence, many strategies, such as band gap (*E_g_*) engineering, introducing luminescent materials, constructing heterostructure, and promoting charge separation efficiency via depositing metal ions, were proposed to settle these issues [8,9,10,11]. Although the photocatalytic behaviors of the semiconductors have been modified to a certain degree with the help of these aforementioned techniques, more efforts should be made to further elevate their photocatalytic activities as well as extend their vivid applications.

To date, different types of photocatalysts, such as CdSe@ZnO composite, carbon nanotube@zeolite nanocomposite, ZnO@SiO_2_ composite, and SrO, have been developed to remove pollutants so as to realize wastewater treatment [12,13,14,15]. In comparison with other semiconductors, bismuth-containing materials are regarded as promising visible-light-driven photocatalysts for pollutant degradation on account of their excellent characteristics of relatively small *E_g_* value, low cost, non-toxicity and controllable morphology [16,17,18,19]. Bi et al. reported that the Bi_4_O_5_I_2_/Bi_5_O_7_I heterojunction can degrade antibiotic tetracycline efficiently under visible light irradiation [20]. Zhang et al. found that these compounds with the chemical expression of Bi*_x_*O*_y_*Cl are able to remove RhB from water under visible light excitation [21]. Moreover, it is also revealed that the BiOX (X = F, Cl, Br, I) particles had the ability to reduce CO_2_ under a simulated solar light source irradiation [22]. These previous findings indicate that the bismuth-containing semiconductors are suitable for photodegrading pollutants. In particular, the interest in BiOX is increasing and they are widely employed in the field of photocatalysis because of their special matlockite structures along with [-X-Bi-O-O-Bi-X] layers, which are characterized by [Bi_2_O_2_]^2+^ slabs interleaved by two halogen ions [23,24,25]. Among them, the photocatalytic activity of BiOBr, which possesses a tetragonal phase with a suitable *E_g_* value of approximately 2.74–2.88 eV, has been intensively studied [26,27]. Nevertheless, previously developed BiOBr particles usually exhibited unsatisfactory photocatalytic behaviors since they needed a long time (i.e., *t* > 100 min) to degrade pollutants upon visible light excitation [28,29,30]. Therefore, some strategies should be carried out to further manipulate the photocatalytic activity of BiOBr compounds.

Stimulated by the aforementioned issues, we selected BiOBr as the research target and the hydrothermal method was applied to synthesize BiOBr particles by using diverse solvents. The phase structure, morphology characteristics, chemical compositions and light harvest capacity of resultant compounds were investigated in detail. Moreover, upon visible light irradiation, the photocatalytic activity of BiOBr particles was explored through analyzing the photodegradation of RhB. Furthermore, various reaction solvents were used to synthesize BiOBr particles so as to reveal their impact on the photocatalytic properties of the designed compounds. Ultimately, based on the trapping experiment and ESR results, we studied the involved mechanism of photocatalysis. 

Inspired by the above research on the properties of bismuth-containing semiconductors, we chose BiOBr as the research object to study its photocatalytic activity for ultrafast degradation of organic molecules (pollutants) under visible light irradiation. BiOBr particles were synthesized by the hydrothermal method in different reaction solvents, and the phase structure, morphological characteristics, chemical composition and light-trapping ability of the obtained compounds were studied in detail. In addition, by analyzing the photodegradation rate of BiOBr particles to RhB under visible light irradiation, the effect of the synthetic conduction on the photocatalytic activity of BiOBr particles was explored. Finally, based on the results of capture experiment and electron spin resonance (*ESR*) measurement, we proposed the photocatalytic mechanism of BiOBr particles under visible light irradiation.

## 2. Results and Discussion

**Figure 1:**
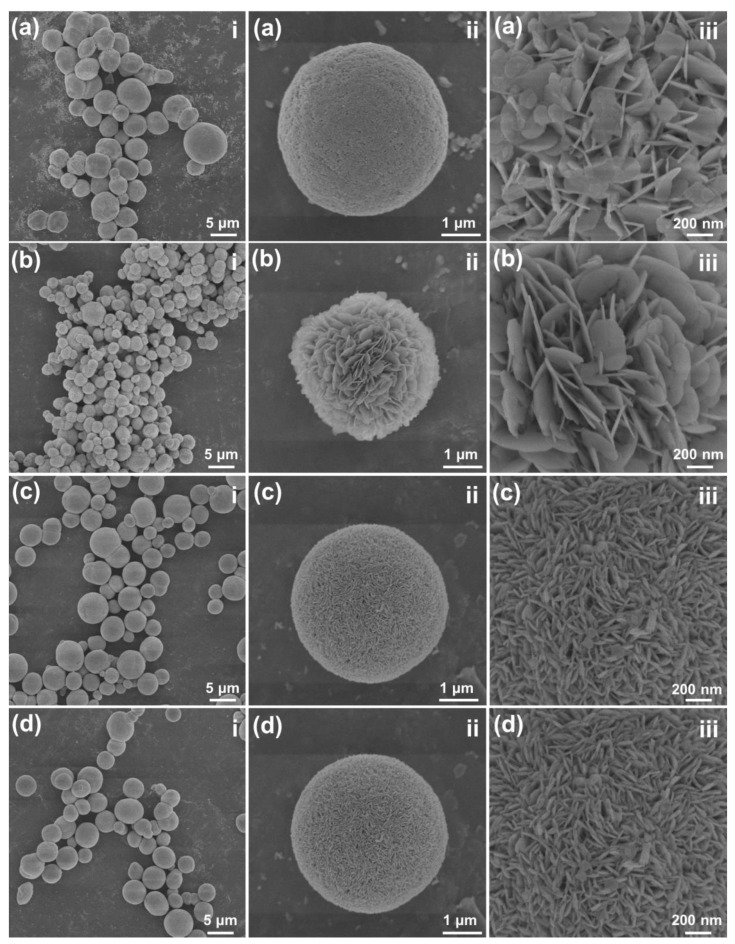
FE-SEM images of BiOBr particles synthesized by using different solvents of (**ai**–**aiii**) EG, (**bi**–**biii**) EG:EtOH = 2:1, (**ci**–**ciii**) EG:EtOH = 1:1, and (**di**–**diii**) EG:EtOH = 1:2.

The X-ray diffraction (XRD) profiles of BiOBr particles, which were prepared by different volume ratios of EG to EtOH, were examined and shown in Figure S1. Clearly, the diffraction profiles of the whole samples are the same and they match well with that of the tetragonal BiOBr (JCPDS#09-0393), implying that the final products exhibit a pure hexagonal phase and the changing of the volume ratio of EG to EtOH hardly impacts the phase compositions of BiOBr particles. Moreover, we utilized the FE-SEM to explore the morphology performance of BiOBr particles synthesized via diverse solvents, as depicted in Figure 1. From the low-magnification FE-SEM images (see Figure 1(ai–di)), it is clear that all of the designed compounds are composed of microsphere. However, when we shift to the high-magnification FE-SEM images, as shown in Figure 1(aii–diii), it is clear that the surfaces of these particles are different. In particular, when the EG content is high, one finds that these spherical particles consist of loose nanosheets, as presented in Figure 1(aiii–biii). Nevertheless, when the EtOH content is increased, it is evident that these microspheres are made of close-knit nanosheets (see Figure 1(ciii–diii)). These findings indicate that the morphology behaviors of final compounds are sensitive to the volume ratio between EG and EtOH, which can further impact their visible-light-driven photocatalytic activities. Furthermore, elemental compositions of resultant samples are detected by EDS. As disclosed in Figure S2a, the developed products comprise Bi, O and Br elements. Additionally, the elemental mapping results demonstrate that these elements are uniformly distributed in the particles (see Figure S2b–d). 

**Figure 2:**
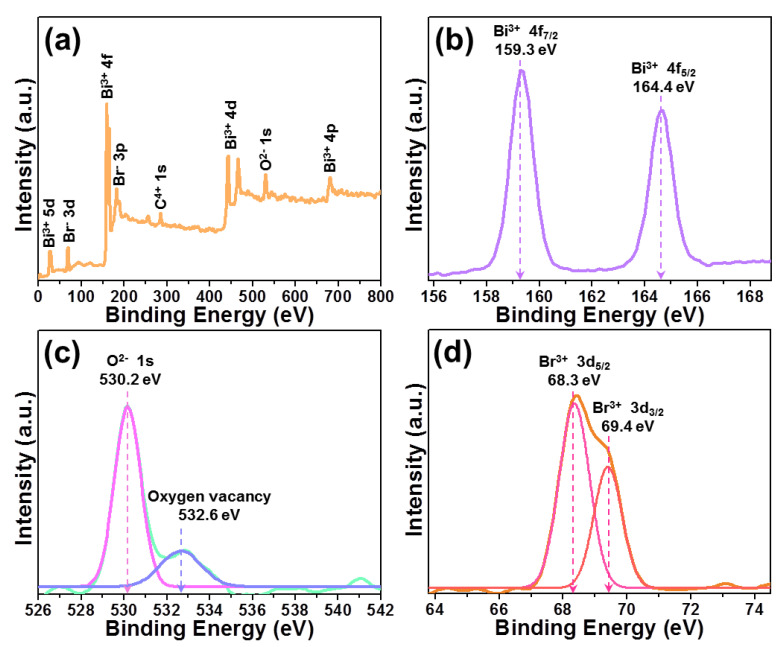
XPS spectra of BiOBr particles. (**a**) Full survey spectrum, (**b**) Bi^3+^ 4f, (**c**) O^2−^ 1s and (**d**) Br^−^ 3d.

For the purpose of further confirming the surface chemical states and compositions of studied samples, the XPS spectra of BiOBr particles were examined and illustrated in Figure 2. According to the full survey spectrum (see Figure 2a), it is obvious that the resultant particles only consist of Bi, Br and O elements. Moreover, the high-resolution XPS spectrum of Bi^3+^ 4f shown in Figure 2b contains two strong bands at 159.3 and 164.4 eV corresponding to Bi^3+^ 4f_7/2_ and Bi^3+^ 4f_5/2_, respectively [9,16]. As for the high-resolution XPS spectrum of O^2−^ 1s (see Figure 2c), one knows that it can be divided into two peaks at approximately 530.2 and 532.6 eV, which are assigned to O^2−^ 1s and oxygen vacancy, respectively [16,31]. Based on previously reported literatures, it is significant that the existence of oxygen vacancy in photocatalysts is benefit for improving their photocatalytic activities since it can promote the light harvest capacity of photocatalysts [16,31]. Figure 2d illustrates the high-resolution XPS spectrum of Br^−^ 3d. Apparently, the detected peak is asymmetric and it is able to be deconvoluted into two peaks at about 68.3 and 69.4 eV corresponding to Br^−^ 3d_5/2_ and Br^−^ 3d_3/2_, respectively [22]. These results indirectly confirm the successful synthesis of BiOBr particles.

As everyone knows, light capture capacity plays an important role in determining the photocatalytic activities of photocatalysts. Herein, we recorded the UV–vis absorption spectra of BiOBr particles, which were synthesized by using various contents of EG and EtOH, as shown in Figure S3. As demonstrated, the resultant samples exhibit an intense absorption in the wavelength range of 200–550 nm, indicating that visible-light-driven photocatalytic activity is able to be realized in the synthesized particles. Note that, the light absorption ability of the studied samples is barely affected by reaction condition. Moreover, the *E_g_* value of a semiconductor is able to be evaluated via the expression of $\alpha hv=A{\left(hv-E_{g}\right)}^{n}$ (herein, *α* denotes the absorbance coefficient, *hv* stands for the photon energy, *A* refers to an coefficient and *n* has several values which are decided by the type of semiconductors) [32,33]. Since BiOBr belongs to an indirect semiconductor, the *n* value is 2. Consequently, with the aid of the above expression as well as the recorded UV–vis absorption spectra, the *E_g_* values of BiOBr particles prepared by different solvents are estimated and shown in Figure S4. Significantly, the *E_g_* values of resultant samples are insensitive to the volume ratio of EG to EtOH, in which *E_g_* equals to 2.75 eV. 

**Figure 3:**
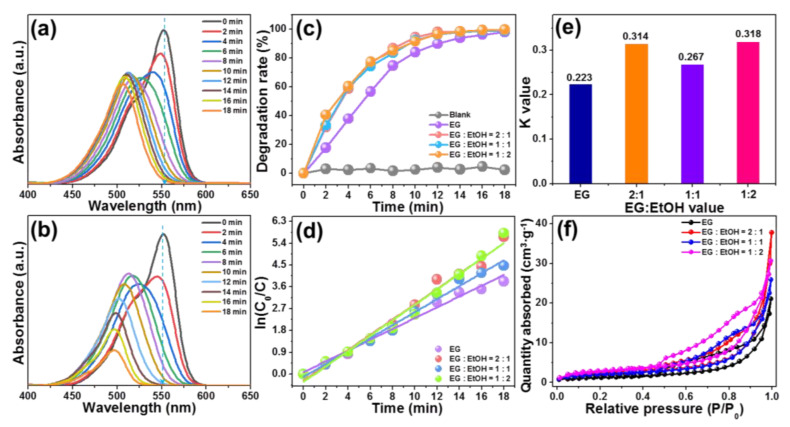
UV–vis absorption spectra of RhB photodegradation in the presence of BiOBr particles prepared by diverse solvents of (**a**) EG and (**b**) EG:EtOH = 1:2. (**c**) Photodegradation efficiency of RhB via the use of synthesized photocatalysts. (**d**) ln(*C*_0_/*C*) versus *t* for the developed particles. (**e**) *K* values of and (**f**) N_2_ absorption–desorption isotherms of BiOBr compounds prepared by different solvents.

To determine the photocatalytic activities of the synthesized BiOBr particles, the visible-light-driven photodegradation of RhB by employing the resultant photocatalysts was performed. The UV–vis absorption spectra of RhB aqueous solution in the presence of designed BiOBr particles, which were prepared by using various solvents of pure EG and EG:EtOH = 1:2, are demonstrated in Figure 3a,b, respectively. As revealed, with the increment of irradiation time, the recorded UV–vis absorption spectra change a lot. In particular, not only the intensity of absorption band of RhB (i.e., at 554 nm) show a downward tendency, but also its position exhibits a blue shift due to the de-ethylation RhB triggered by attacking the reactive oxygen species on N-ethyl group [9,16], leading to the generation of intermediate products. Similar phenomena are also gained other BiOBr particles prepared by different solvents of EG:EtOH = 2:1 and EG:EtOH = 1:1, as presented in Figure S5. These findings directly reflect that the RhB dye can be decomposed by the developed BiOBr particles excited at visible light.

For the sake of quantitatively describing the photodegradation capacity of the synthesized compounds, the following function is applied to evaluate the photodegradation efficiency of RhB via using of BiOBr particles, as defined below [34,35]:

(1) $$\mathrm{Degradation}\;\mathrm{rate}=\left(1-\frac{C}{C_{0}}\right)\times 100\%$$

where *C* stands for the content of RhB at irradiation time *t* and *C*_0_ is regarded as the initial content of RhB. Via utilization of the measured UV–vis absorption spectra and Equation (1), the time-dependent degradation rate of RhB by resultant samples was calculated and shown in Figure 3c. As disclosed, the obtained values are greatly impacted by the irradiation time. In particular, for the BiOBr particles prepared by EG, it takes approximately 18 min to totally degrade the RhB upon visible light excitation, while for other samples prepared by the mixed solutions of EG and EtOH, they can finish the photodegradation process within 12 min when excited by visible light. Moreover, a blank experiment, in which the designed BiOBr particles were not added, was also performed so as to clarify the self-decomposition of RhB under visible light irradiation. From Figure 3c, it is obvious that RhB does not degrade after exposing to visible light for 18 min. These results suggest that the BiOBr particles have splendid visible-light-driven photocatalytic activity and it is able to be tuned via manipulating the volume ratio of EG to EtOH. To explain the involved photocatalytic mechanism, it is necessary to investigate the reaction kinetic (*K*) and it can be evaluated through the following function [36,37]:

(2) $$\ln\left(\frac{C_{0}}{C}\right)=Kt$$

Herein, the exact meanings of *C* and *C*_0_ have been defined in Equation (1), *K* is assigned to the first-order rate coefficient. The relation between ln(*C*_0_/*C*) and *t* is shown in Figure 3d. Through linearly fitting, the experimental data are fitted, in which *K* value equals to the slope of fitted line. As depicted in Figure 3e, it is clear that the *K* values of BiOBr particles synthesized by diverse solutions of EG, EG:EtOH = 2:1, EG:EtOH = 1:1 and EG:EtOH = 1:2 are decided to be 0.223, 0.314, 0.267 and 0.318 min^−1^, respectively. Evidently, the *K* value of BiOBr particles prepared by the mixed solvent of EG:EtOH = 1:2 is larger than other samples, further implying that the photocatalytic activity of BiOBr particles is able to be adjusted by changing the reaction solvents with different the volume ratio of EG to EtOH in the preparing process. As discussed above, although all the synthesized compounds have similar absorption capacity, their morphology properties are different, which may be the key factor to impact the photocatalytic activity of resultant photocatalysts. In order to verify this conjecture, we estimated the specific surface area of the developed compounds by the N_2_ absorption–desorption isotherms, as depicted in Figure 3f. Clearly, these recorded curves show typical IV hysteresis loop (see Figure 3f), implying the existence of mesoporous structures in the studied samples. Furthermore, the specific surface areas of BiOBr particles synthesized by various solvents of EG, EG:EtOH = 2:1, EG:EtOH = 1:1 and EG:EtOH = 1:2 are 5.625, 8.021, 7.378 and 10.048 m^2^/g, respectively, as displayed in Table S1. In a general case, larger specific surface area is able to support more surface active sites which are benefit to transporting reactants, leading to the enhanced photocatalytic activity [38]. Thereby, excited by visible light, the BiOBr particles prepared by the solvent of EG:EtOH = 1:2 exhibit the optimal photocatalytic activity compared with other as-prepared compounds.

**Figure 4:**
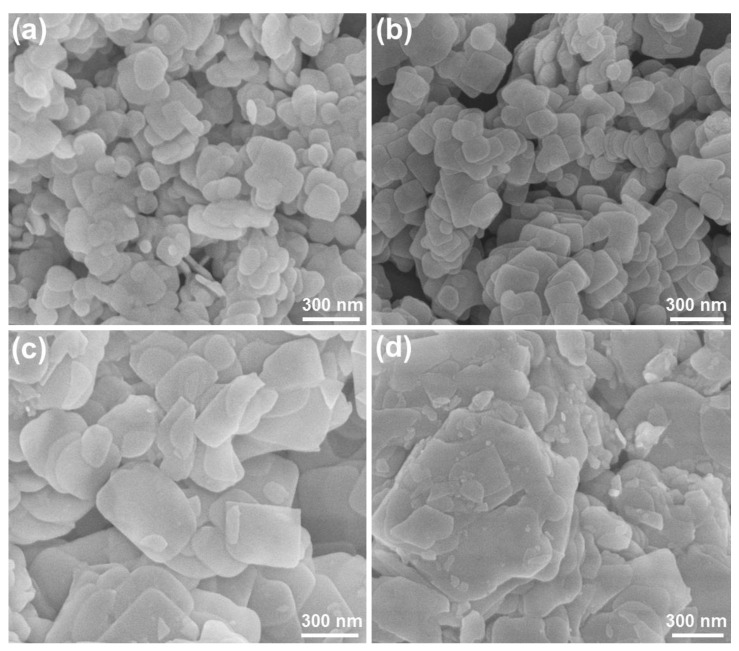
FE-SEM images of BiOBr particles synthesized by using different solvents of (**a**) EG:H_2_O = 2:1, (**b**) EG:H_2_O = 1:1, (**c**) EG:H_2_O = 1:2, and (**d**) H_2_O.

Aside from utilizing the mixed solvent of EG and EtOH to prepare BiOBr, we also tried to employ other solvents (i.e., EG and H_2_O) to synthesize BiOBr particles and explore their visible-light-driven photocatalytic activities. Via the use of different contents of EG and H_2_O, we synthesized series of BiOBr particles and their crystal structures were confirmed by XRD, as shown in Figure S6. Significantly, in spite of increasing the H_2_O content, the phase structure of the resultant compounds is not changed and all the samples exhibit a single hexagonal phase (see Figure S6). Further, the light absorption abilities of BiOBr particles prepared by different volume ratios of EG to H_2_O were also verified through the UV–vis absorption spectrum, as demonstrated in Figure S7. Clearly, the synthesized samples exhibit similar absorption spectra with an absorption edge at approximately 500 nm, suggesting that the light harvest ability of BiOBr particles is scarcely influenced by adjusting the volume ratio of EG to H_2_O. Furthermore, the FE-SEM images of BiOBr particles synthesized via the use of different mixed solvents are presented in Figure 4. From Figure 4a, one finds that the studied products are made up of small-sized nanoplates with an average size of approximately 170 nm when the volume ratio of EG to H_2_O is 2:1. However, when the volume ratio of EG to H_2_O is shifted to 1:1, relatively uniform thin nanoplates with a size of approximately 300 nm are gained in the resultant compounds (see Figure 4b). With further increasing the volume ratio of EG to H_2_O to 1:2, one can only obtain large nanoplates in the designed compounds, as illustrated in Figure 4c. Notably, when H_2_O is used to prepare the BiOBr particles, it is demonstrated in Figure 4d that the final compounds are composed of condensed microparticles. These aforementioned results confirm that the manipulation of the volume ratio of EG to H_2_O has little impact on the phase structure and light harvest ability of BiOBr particles, whereas it can influence the morphology of the synthesized compounds.

**Figure 5:**
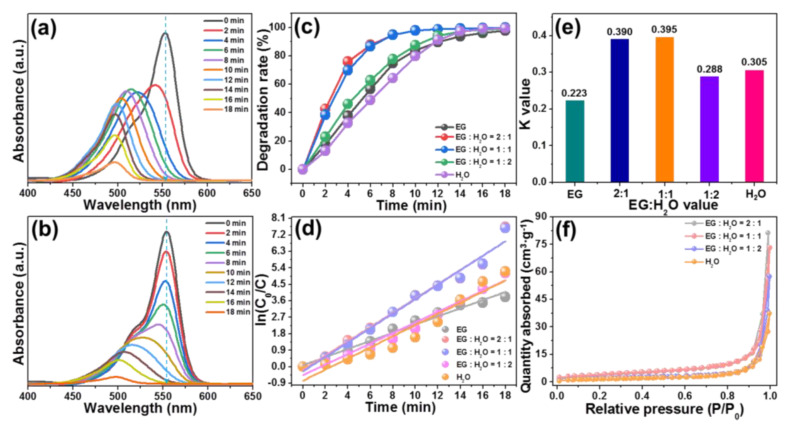
UV–vis absorption spectra of RhB photodegradation in the presence of BiOBr particles prepared by diverse solvents of (**a**) EG:H_2_O = 2:1 and (**b**) H_2_O. (**c**) Photodegradation efficiency of RhB via the use of synthesized photocatalysts. (**d**) ln(*C*_0_/*C*) versus *t* for the developed particles. (**e**) *K* values of and (**f**) N_2_ absorption–desorption isotherms of BiOBr compounds prepared by different solvents.

Though studying the photodegradation of RhB by employing the developed BiOBr particles under visible light irradiation, the photocatalytic activities of the resultant compounds are investigated. The UV–vis absorption spectra of RhB photodegradation in the presence of BiOBr particles prepared by diverse solvents of EG:H_2_O = 2:1 and H_2_O are shown in Figure 5a,b, respectively. Evidently, with increasing the irradiation time, the intensity of absorption band of RhB is reduced gradually due to the decomposition of RhB under visible light excitation. Aside from the reduced intensity, blue shift is also observed in the absorption band. Note that, this kind of situation is not changed when other BiOBr particles, which were synthesized by using various solvents of EG:H_2_O = 1:1 and EG:H_2_O = 1:2, are used, as described in Figure S8. The time-dependent degradation rate of RhB by using the resultant photocatalysts is depicted in Figure 5c. As presented, these BiOBr particles, which were prepared by using the mixed solutions of EG:H_2_O = 1:2 or H_2_O, need 16 min to photodegrade RhB under visible light irradiation, whereas other BiOBr particles, which were synthesized by using the mixed solutions of EG:H_2_O = 2:1 or EG:H_2_O = 1:1, only need 10 min to fully decompose RhB. Note that, these BiOBr particles prepared by mixed solvents of EG and H_2_O show better photocatalytic activities than the BiOBr particles synthesized by using EG, in which it takes 18 min to decompose RhB under visible light irradiation. Further, compared with previously developed BiOBr particles [28,29,30], our sample also presents much better visible-light-triggered photocatalytic activities. Furthermore, for the aim of achieving the *K* value of the involved photocatalytic process, the relation between ln(*C*_0_/*C*) and *t* was plotted, as described in Figure 5d. Apparently, these experimental data can be linearly fitted. As displayed in Figure 5e, the *K* values of BiOBr particles prepared by diverse solutions of EG, EG:H_2_O = 2:1, EG:H_2_O = 1:1, EG:H_2_O = 1:2 and H_2_O are 0.223, 0.390, 0.395, 0.288 and 0.305, respectively. In addition, the N_2_ adsorption–desorption isotherm curves of the resultant BiOBr particles synthesized by different solutions were tested and demonstrated in Figure 5f. It can be seen Figure 5f that these tested curves contain significant hysteresis loops, implying that the pores are existed in the resultant particles. The calculated values of the specific surface areas of synthesized BiOBr particles are listed in Table S1. Clearly, when the volume ratio of EG to H_2_O is changed, the specific surface areas of final products are totally different (see Table S1), which might be responsible for the modified photocatalytic activity. These results further prove that the photocatalytic activity of BiOBr particles is able to be manipulated by controlling the volume ratio of EG to H_2_O. 

**Figure 6:**
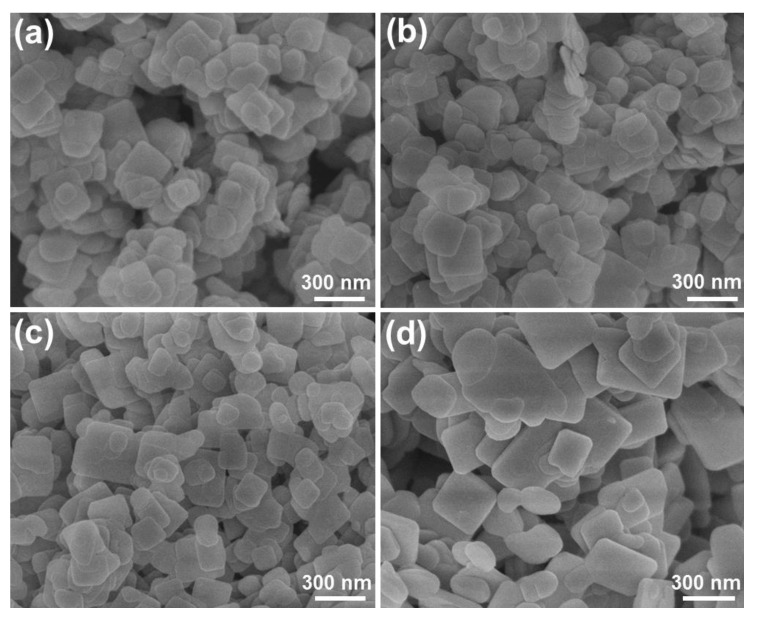
FE-SEM images of BiOBr parties prepared by various heat treatment temperatures of (**a**) 473 K, (**b**) 573 K, (**c**) 673 K and (**d**) 773 K.

Except for the reaction solvent, can the photocatalytic features of BiOBr particles be impacted by the heating temperature since it has significant influence of the basic properties (i.e., morphology, phase structure, crystalline, etc.) of final products? To figure out this doubt, heat treatment with different sintering temperatures was performed, in which the initial BiOBr nanoplates prepared by the hydrothermal method with the solvent of EG:H_2_O = 1:1 were adopted as the research objects. The XRD patterns of BiOBr nanoplates treated by different temperatures were tested and shown in Figure S9. It is clear that all the studied samples display the same diffraction profile and these detected diffraction peaks can be indexed by the standard tetragonal BiOBr (JCPDS#09-0393), revealing that the phase structure of final compounds is hardly impacted by the heating temperature in the interest of our range. Furthermore, the FE-SEM images of the resultant products are summarized in Figure 6 so as to identify the relation between the sintering temperature and morphology. Apparently, when the sintering temperature is low (i.e., T ≤ 673 K), the morphology of the prepared compounds is slightly changed, in which thin nanoplates are observed (see Figure 6a–c), whereas large-sized nanoplates are observed in the synthesized samples prepared by high sintering temperature (i.e., T = 773 K), as illustrated in Figure 6d. In particular, when the sintering temperature is increased to 773 K, not only the size of nanoplates is enlarged but also its thickness is increased. On the other hand, via the use of UV–vis absorption spectra, we also studied the light harvest capacity of the resultant BiOBr particles. As presented in Figure S10, there are little changes in the absorption spectra as well as the absorption edge of the final products even though the heat treatment is carried out, manifesting that their light capture ability is insensitive to the sintering temperature. These above findings imply that the sintering temperature does not have the ability to influence the phase structure and light harvest ability of BiOBr particles, whereas it is able to alter the morphology of the studied samples, which may affect their photocatalytic activities in turn. 

**Figure 7:**
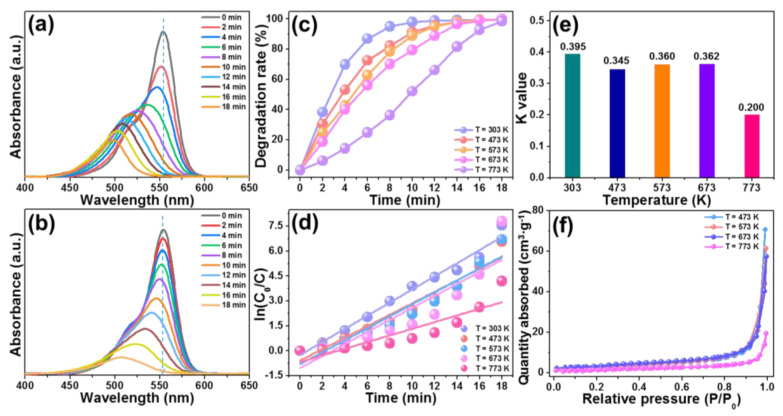
UV–vis absorption spectra of RhB photodegradation in the presence of BiOBr particles obtained by different heat treatment temperatures of (**a**) T = 473 K and (**b**) T = 773 K. (**c**) Photodegradation efficiency of RhB via the use of synthesized photocatalysts. (**d**) ln(*C*_0_/*C*) versus *t* for the developed particles. (**e**) *K* values and (**f**) N_2_ absorption–desorption isotherms of BiOBr compounds prepared by different solvents.

For the aim of digging out the influence of heat treatment on the photocatalytic activities of the studied nanopolates, the visible-light-driven photodegradation of RhB via using the obtained BiOBr particles was performed. The UV–vis absorption spectra of RhB photodegradation in the presence of BiOBr particles treated by diverse sintering temperatures of 473 and 773 K are depicted in Figure 7a,b, respectively. Significantly, the intensity of the characterize absorption band of RhB (i.e., at 554 nm) shows a downward tendency as the irradiation time increases, suggesting the degradation of RhB is realized by using the obtained BiOBr particles under visible light irradiation. Moreover, we also find that the absorption band exhibits a blue shift with increasing the irradiation time. Herein, the same phenomenon is also seen other BiOBr compounds prepared by different sintering temperatures, as shown in Figure S11. To get deeper insight into the photocatalytic properties of the resultant nanoplates, the time-dependent degradation rate was calculated by utilizing Equation (1) and the corresponding results are demonstrated in Figure 7c. As revealed, with the increment of irradiation time, the degradation rates increase gradually, in which their maximum values are achieved at different irradiation time. Note that, the BiOBr particles without heat treatment (i.e., T = 303 K) only use 10 min to degrade RhB under visible light excitation, while more time (i.e., *t* ≥ 14 min) is required when the heat treatment is produced (see Figure 7c), indicating that the photocatalytic activities of BiOBr nanoplates are restrained by employing the heat treatment engineering. The plots of ln(*C*_0_/*C*) vs. *t* for the obtained BiOBr particles are presented in Figure 7d so as to achieve their corresponding *K* values. Through linearly fitting these experimental data, the *K* values (i.e., slope) of BiOBr particles without heating and with heating at 473, 573, 673 and 773 K are decided to be 0.395, 0.345, 0.360, 0.362, and 0.200, as presented in Figure 7e, respectively. In comparison, the final products without heat treatment have larger *K* value, further demonstrating that the extra heat treatment is able to suppress the photocatalytic behaviors of BiOBr particles. In order to figure out the probable reason for this observed phenomenon, the N_2_ adsorption–desorption isotherms of these BiOBr nanoplates were measured so as to evaluate their specific surface area, as displayed in Figure 7f and Table S2. It is clear that the surface area of resultant nanoplates is reduced when the heat treatment is employed. Due to the relatively smaller specific surface area, depressed photocatalytic activities are observed in the studied samples.

**Figure 8:**
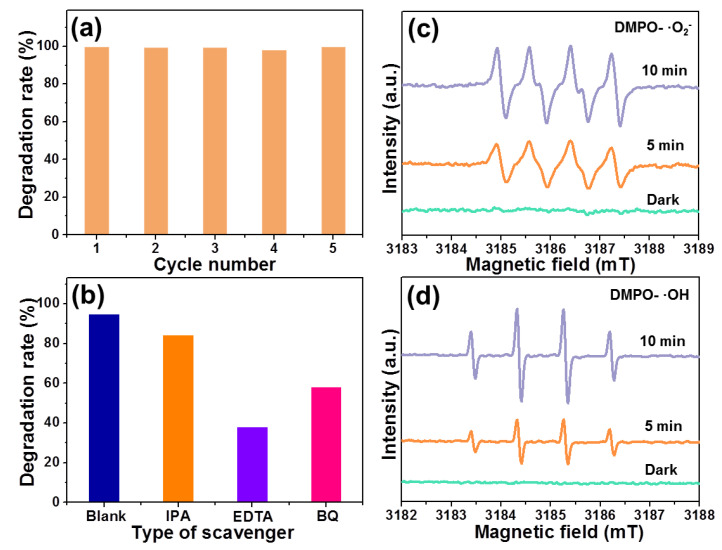
(**a**) Recycling photodegradation performance of RhB in the presence of synthesized BiOBr particles upon visible light irradiation. (**b**) Photodegradation efficiency of RhB via the use of BiOBr with different scavengers. ESR spectra of (**c**) DMPO-·O_2_^−^ and (**d**) DMPO-·OH adducts of BiOBr particles without and with visible light irradiation.

When the developed photocatalysts are used in real life, they should present high stability and recyclability. Herein, upon visible light irradiation, a cycle experiment by exploring RhB photodegradation via the use of resultant particles was put forward and the corresponding results are shown in Figure 8a. It is obvious that the photocatalytic activity of BiOBr particles does not decrease even after being reused for five times. Moreover, it is clear that phase structure of the studied samples is not changed after the photodegradation process, as confirmed by the XRD pattern demonstrated in Figure S12. These final results suggest that the designed photocatalysts have splendid durability. As discussed above, the synthesized BiOBr particles have excellent photocatalytic activities under visible light irradiation. Thus, it is necessary to explore its visible-light-driven photocatalytic mechanism. Generally, there are three types of active species, namely, h^+^, ·O_2_^−^ and ·OH, that can contribute to the photocatalytic process. Thereby, we did a series of trapping experiments, in which BQ, IPA and EDTA were employed as scavengers to capture ·O_2_^−^, ·OH and h^+^, respectively. As indicated in Figure 6b, when BQ and EDTA were added, the degradation efficiencies of BiOBr particles are significantly reduced, implying that the involved visible-light-driven photocatalytic mechanism is primarily contributed to by ·O_2_^−^ and h^+^ active species. In comparison, the photocatalytic activity of BiOBr particles does not decrease so much when IPA is introduced, as described in Figure 8b, suggesting that ·OH also plays a role in the photocatalytic process. In order to further determine the active species during the photodegradation RhB process, the ESR experiments were carried out and the corresponding results are shown in Figure 8c,d). When the samples are put in the dark environment, the signals of ·OH and ·O_2_^−^ are not detected, whereas the sharp peaks originating from ·OH and ·O_2_^−^ are observed upon irradiating of visible light. Moreover, the intensities of the DMPO-·OH and DMPO-·O_2_^−^ signals are also revealed to be improved with the increase in irradiation time. According to the ESR results, one can conclude that the active species of ·OH and ·O_2_^−^ can be formed during the visible-light-driven photocatalytic process, which matches well with results achieved from the trapping experiments. 

**Figure 9:**
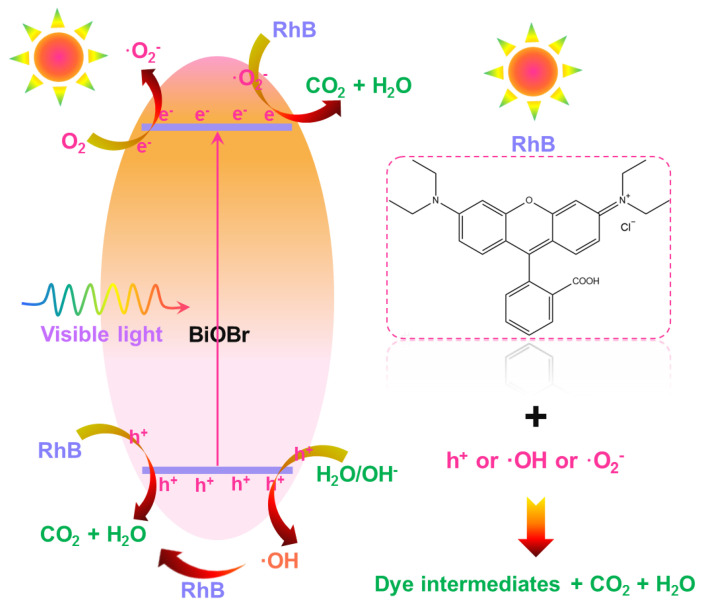
Possible visible-light-driven photocatalytic mechanism of developed BiOBr particles.

As confirmed above, the synthesized BiOBr particles have good photocatalytic activity under visible light irradiation, where the active species of h^+^, ·OH and ·O_2_^−^ are responsible for the involved photocatalytic mechanism. In order to intuitively describe the visible-light-driven photocatalytic mechanism, the possible photocatalytic processes are drawn and shown in Figure 9. As demonstrated, when BiOBr particles are exposed to visible light, electron (e^−^) located at the valence band (VB) of BiOBr would be pumped to the conduction band (CB) of BiOBr, resulting in the formation of hole–electron pairs. In particular, electrons populated at CB will react with oxygen so as to form the ·O_2_^−^ active species which can further react with RhB, leading to the photodegradation of RhB. In comparison, h^+^ located at VB can take participate in two different processes, namely, h^+^ can not only directly react with RhB, resulting in the photodegradation of RhB, but also is able to react with H_2_O/OH^−^ couple to generate ·OH active species and it can efficiently decompose RhB, as presented in Figure 9. Furthermore, the existed oxygen vacancy in the studied samples is also benefit to capturing oxygen and harvesting electron, which can promote the formation of ·O_2_^−^ active species, leading to the excellent photocatalytic activity [27]. 

Ultimately, via the use of the following expressions, we summarized the photocatalytic processes of the studied samples:

BiOBr + *hv* (i.e., visible light) → h^+^ + e^−^ (3)

O_2_ + e^−^ → ·O_2_^−^ (4)

h^+^ + H_2_O/OH^−^ → ·OH (5)

h^+^ or ·O_2_^−^ or ·OH + RhB → CO_2_ +H_2_O + intermediate products (6)

Through these aforementioned processes, the developed BiOBr particles possess splendid photocatalytic activities and can decompose RhB within a short time upon visible light irradiation.

## 3. Experimental Process

### 3.1. Hydrothermal Synthesis of BiOBr Particles

The hydrothermal method was adopted to prepare BiOBr particles. In a typical process, we weighed 1 mmol of Bi(NO_3_)_3_·5H_2_O and 1 mmol of hexadecyl trimethyl ammonium bromide (CTAB) via an electronic balance, and then put them in a beaker containing 30 mL of ethylene glycol (EG). After mixing for 30 min, the mixture was transferred to an autoclave (50 mL) and heated at 120 °C for 2 h. When the reaction was completed, the BiOBr particles were synthesized and collected after washing by ethanol (EtOH) and deionized water several times, and then drying at 100 °C for 4 h. Notably, to synthesize BiOBr particles by using different reaction solvents, all the processes were the same as aforementioned except that the EG was replaced by other solvents including EG:EtOH = 2:1, EG:EtOH = 1:1, EG:EtOH = 1:2, EG:H_2_O = 2:1, EG:H_2_O = 1:1, and EG:H_2_O = 1:2 and H_2_O, where the ratio stands for volume ratio and total volumes of solvents were all 30 mL. Additionally, we selected these BiOBr particles prepared by using the solvent of EG:H_2_O = 1:1 to be used in the heat treatment process, in which the sintering temperature was fixed at 473, 573, 673 and 773 K. Note that, the calcination time was 4 h. 

### 3.2. Characterization

The X-ray diffractometer (Bruker D8 Advance, Bruker, Billerica, MA, USA), where the Cu k*α* is used as the irradiation source, was adopted to determine the crystal structure. The morphology characteristics of the designed particles were identified by a field-emission scanning electron microscopy (FE-SEM; Hitachi SU-70, Hitachi High-Tech, Tokyo, Japan) equipped with energy dispersive X-ray spectroscopy (EDS). The chemical components of the final products were examined by a multifunctional imaging electron spectrometer (Thermo ESCLAB 250 XI, Thermo Fisher Scientific, Waltham, MA, USA). Via the use of a surface area analyzer (Micromeritics ASAP2460, Micromeritics, Norcross, GA, USA) by N_2_ adsorption–desorption, the specific surface areas of photocatalysts were explored. 

### 3.3. Photocatalytic Measurement 

Excited by visible light, the photocatalytic performance of the designed particles was examined through studying RhB photodegradation. In this work, we used a 500 W Xe lamp attached with a cutoff filter (λ ≥ 400 nm) as the lighting source. In terms of the photocatalytic experiment, we weighed a certain amount of BiOBr (50 mg) particles and added them into 30 mL of RhB aqueous solution (10 mg/L). Prior to achieving the adsorption–desorption equilibrium, the mixture was kept in a dark environment and stirred for 30 min. After that, the mixture was exposed to visible light. At 2 min intervals, we took 3 mL of aqueous solution and removed the photocatalysts by centrifugation. Herein, the photocatalytic time was 18 min. Finally, the UV–vis absorption spectra of the supernatants were recorded by a spectrophotometer (Cary 500 UV-Vis). Notably, for the purpose of uncovering the recyclability and stability of the final compounds, the powders were washed by deionized water to perform the next cycle experiment. 

### 3.4. Active Species Trapping and ESR Experiment

Isopropanol (IPA; 1 mmol), ethylenediaminetetraacetic acid (EDTA; 1 mmol) and benzoquinone (BQ; 1 mmol) are used to capture h^+^,··O_2_^−^ and··OH active species, respectively. The process of the trapping experiment is same as that of the photocatalytic experiments. In addition, to detect the signal intensities of ·O_2_^−^ and ·OH active substances, we used the 5,5-dimethyl-1-pyrroline-N-oxide (DMPO) as a radical scavenger. With the aid of an electron paramagnetic resonance spectrometer (JES FA200), the ERS spectra were measured.

## 4. Conclusions

In summary, using the different reaction conditions, BiOBr particles with excellent photocatalytic activities were controllably prepared. Upon visible light irradiation, the synthesized BiOBr particles can degrade RhB rapidly and its photocatalytic activities can be manipulated via adjusting the constituent content in the reaction solvent. When reaction solvents with different volume ratios of EG to EtOH were employed, all the resultant samples consisted of microspheres. In particular for the reaction solvent of EG:EtOH = 1:2, the synthesized BiOBr particles can completely decompose RhB (10 mg/L) within 14 min under visible light irradiation. Furthermore, when the reaction solvents are changed to mixtures containing EG and H_2_O, the final compounds also exhibit modified photocatalytic activities. Compared with other samples, BiOBr particles prepared by utilizing the solvent of EG:H_2_O = 1:1 possess the best photocatalytic properties and it only needs 10 min to fully decompose RhB under visible light irradiation, with a *K* value of 0.395. Additionally, when the heat treatment is carried out, depressed photocatalytic activities are obtained in the resultant BiOBr particles. Further, both the trapping and ESR results confirm that the visible-light-driven photocatalytic mechanism of BiOBr particles is contributed to by h^+^, ·OH and ·O_2_^−^ active species. These results indicate that the photocatalytic activity of BiOBr particles driven by visible light can be effectively regulated by BiOBr particles synthesized with different reaction conditions. 

## Data Availability

All data included in this study are available upon request by contact with the first author.

## Supplementary Materials

The following supporting information can be downloaded at: https://www.mdpi.com/article/10.3390/molecules28145558/s1. Figure S1. XRD patterns of BiOBr particles synthesized by using different ratios of EG and EtOH. Figure S2. (a) EDS spectrum and (b–d) Elemental mapping results of BiOBr particles. Inset of (a) shows the FE-SEM image for EDS measurement. Figure S3. UV-vis absorption spectra of BiOBr compounds synthesized by using different solvents of (a) EG, (b) EG:EtOH = 2:1, (c) EG:EtOH = 1:1 and (d) EG:EtOH = 1:2. Figure S4. Calculated Eg values of BiOBr compounds, which were synthesized by using different solvents of (a) EG, (b) EG:EtOH = 2:1, (c) EG:EtOH = 1:1 and (d) EG:EtOH = 1:2, based on Kubelka-Munk function. Figure S5. UV-vis absorption spectra of RhB photodegradation in the presence of BiOBr particles prepared by diverse solvents of (a) EG:EtOH = 2:1 and (b) EG:EtOH = 1:1. Figure S6. XRD patterns of BiOBr particles synthesized by using different ratios of EG and H_2_O. Figure S7. UV-vis absorption spectra of BiOBr compounds synthesized by using different solvents of (a) EG:H_2_O = 2:1, (b) EG: H_2_O = 1:1, (c) EG: H_2_O = 1:2 and (d) H_2_O. Figure S8. UV-vis absorption spectra of RhB photodegradation in the presence of BiOBr particles prepared by diverse solvents of (a) EG:H_2_O = 1:1 and (b) EG:H_2_O = 1:2. Figure S9. XRD patterns of BiOBr particles under various heat treatment temperatures. Figure S10. UV-vis absorption spectra of BiOBr particles prepared by various heat treatment temperatures of (a) T = 473 K, (b) T = 573 K, (c) T = 673 K and (d) T = 773 K. Figure S11. UV-vis absorption spectra of RhB photodegradation in the presence of BiOBr particles synthesized by diverse heating temperatures of (a) T = 573 K and (b) T = 673 K. Figure S12. XRD pattern of BiOBr particles after the photodegradation process. Table S1. Specific surface area of BiOBr particles synthesized by different reaction conditions. Table S2. Specific surface area of BiOBr particles synthesized by different sintering temperatures.
