# Supplementary material for: Controlled Synthesis and Visible-Light-Driven Photocatalytic Activity of BiOBr Particles for Ultrafast Degradation of Pollutants
Source: Molecules. 2023 Jul 20;28(14):5558. doi: 10.3390/molecules28145558 (PMC10384163; doi:10.3390/molecules28145558)
Supplement: Supplementary file 1 [file molecules-28-05558-s001.zip › molecules-2459965-supplementary.pdf]

Controlled Synthesis and Visible-Light-Driven Photocatalytic Activity of BiOBr Particles for Ultrafast Degradation of Pollutants

Xiaohui Ji [1,2], Chen Li [1,2], Junhai Liu [1,2], Tianlei Zhang [1,2], Yue Yang [3], Ruijin Yu [3] and Xuegang Luo [1,4,*]

[1] School of Environment and Resource, Southwest University of Science and Technology, Mianyang 621010, China; snut_xiaohuiji@163.com (X.J.); leechen_317@126.com (C.L.); iamliujunhai@126.com (J.L.); ztianlei88@163.com (T.Z.)
[2] Shaanxi Province Key Laboratory of Catalysis, School of Chemistry and Environmental Science, Shaanxi University of Technology, Hanzhong 723001, China
[3] College of Chemistry & Pharmacy, Northwest A&F University, Yangling 712100, China; rrysdyx@163.com (Y.Y.); yuruijin@nwsuaf.edu.cn (R.Y.)
[4] School of Life Science and Engineering, Southwest University of Science and Technology, Mianyang 621010, China
* Correspondence: lxg@swust.edu.cn

Table S1. Specific surface area of BiOBr particles synthesized by different reaction conditions.

Preparation condition	Surface area (m^2/g)
EG	5.265
EG : EtOH = 2:1	8.021
EG : EtOH = 1:1	7.387
EG : EtOH = 1:2	10.048
EG : H_2O = 2:1	14.011
EG : H_2O = 1:1	14.611
EG : H_2O = 1:2	7.534
H_2O	5.844

Table S2. Specific surface area of BiOBr particles synthesized by different sintering temperatures.

Sintering temperature	Surface area (m^2/g)
303 K	14.611
473 K	10.081

573 K	11.607
673 K	11.735
773 K	5.132

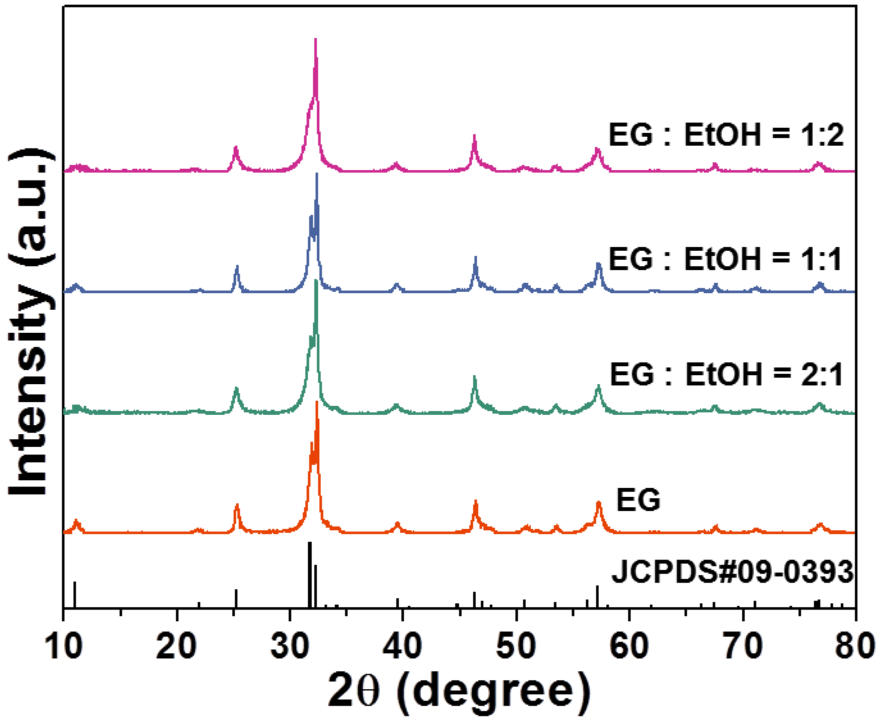

Figure S1. XRD patterns of BiOBr particles synthesized by using different ratios of EG and EtOH.

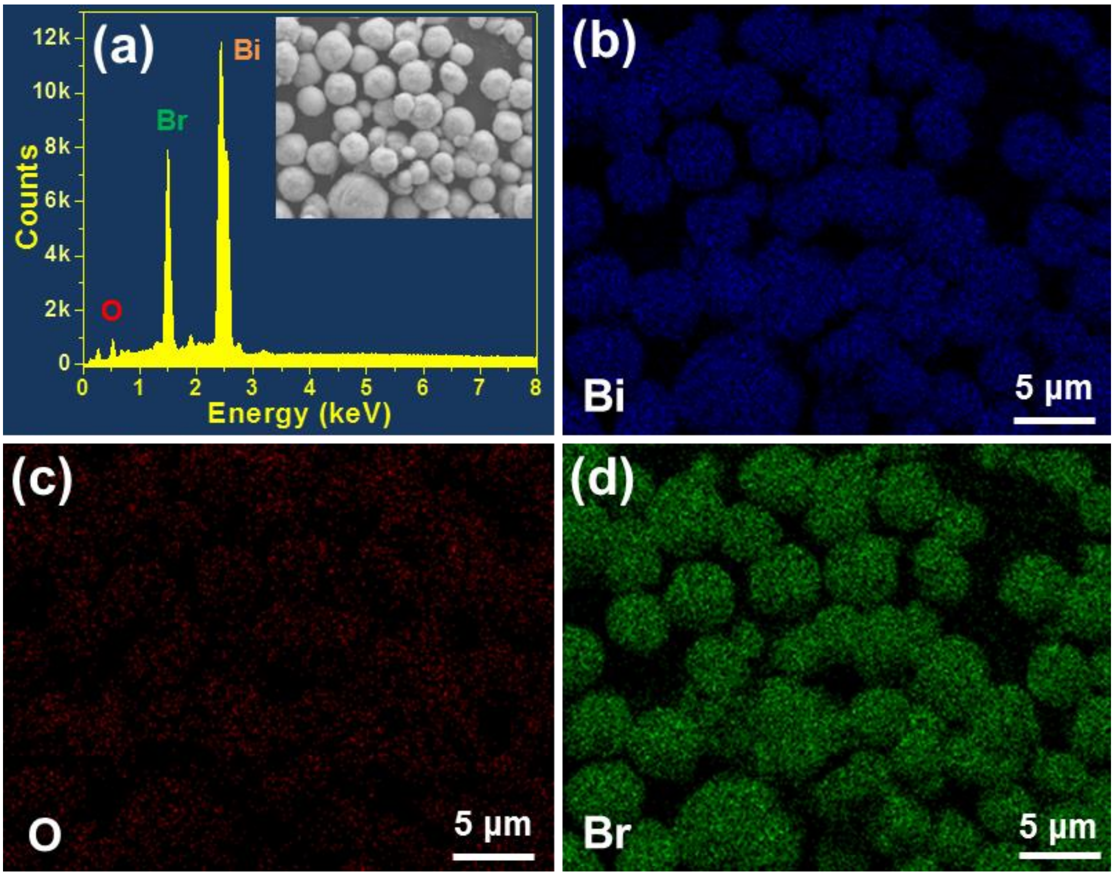

Figure S2. (a) EDS spectrum and (b)-(d) Elemental mapping results of BiOBr particles. Inset of (a) shows the FE-SEM image for EDS measurement.

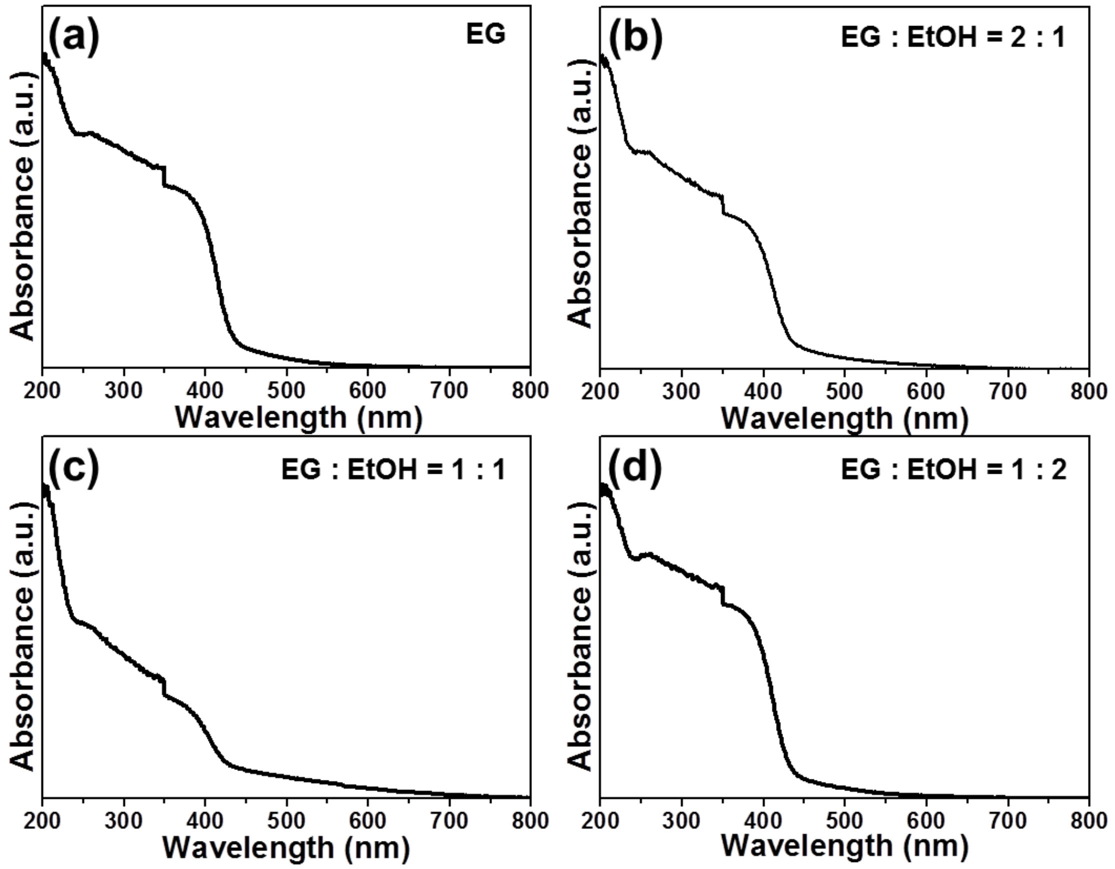

Figure S3. UV-vis absorption spectra of BiOBr compounds synthesized by using different solvents of (a) EG, (b) EG:EtOH = 2:1, (c) EG:EtOH = 1:1 and (d) EG:EtOH = 1:2.

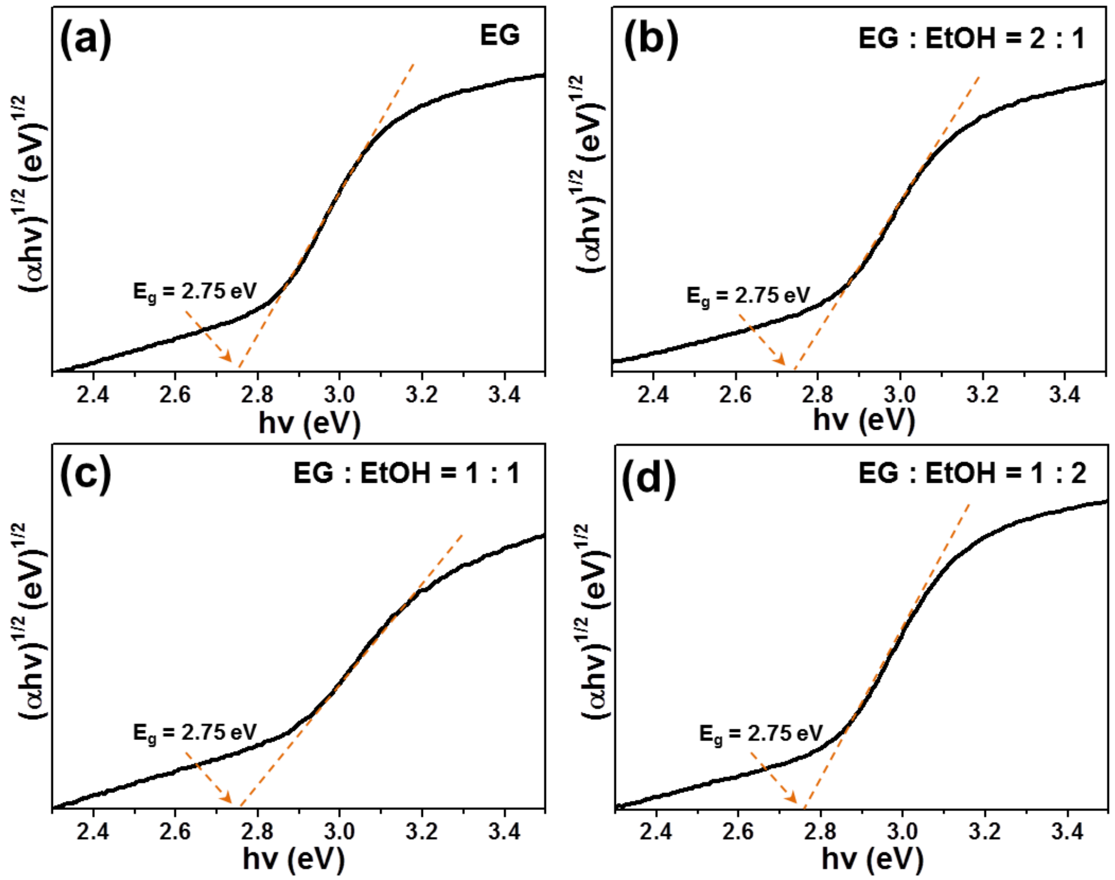

Figure S4. Calculated E_g values of BiOBr compounds, which were synthesized by using different solvents of (a) EG, (b) EG:EtOH = 2:1, (c) EG:EtOH = 1:1 and (d) EG:EtOH = 1:2, based on Kubelka-Munk function.

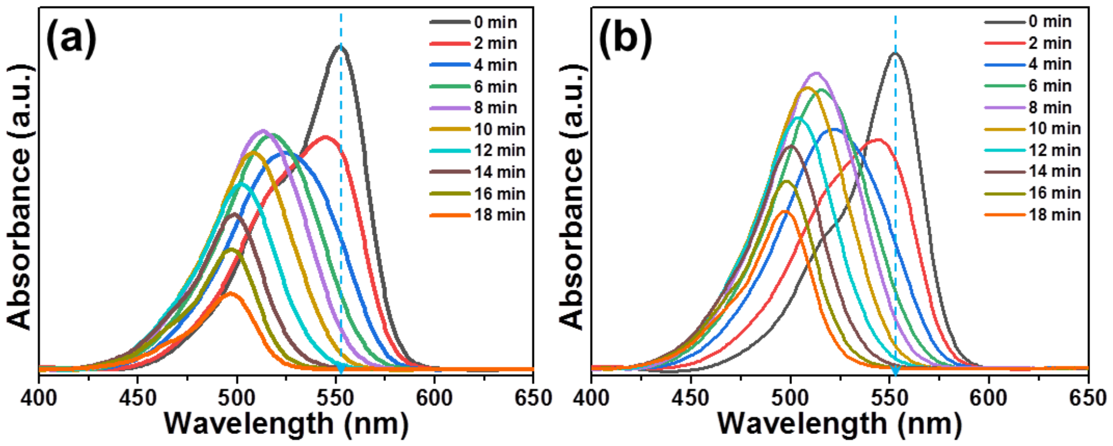

Figure S5. UV-vis absorption spectra of RhB photodegradation in the presence of BiOBr particles prepared by diverse solvents of (a) EG:EtOH = 2:1 and (b) EG:EtOH = 1:1.

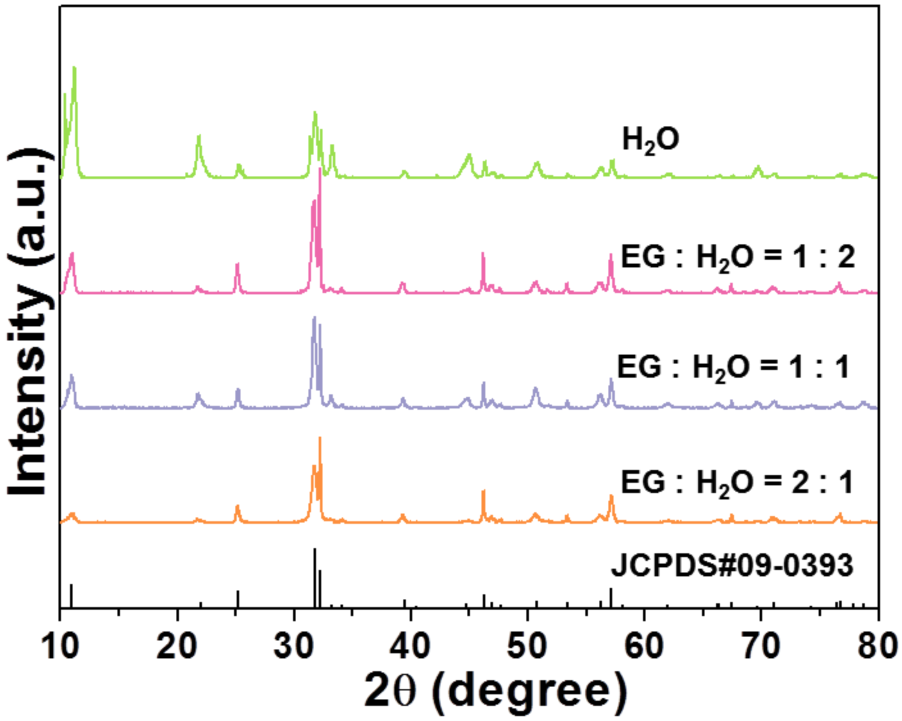

Figure S6. XRD patterns of BiOBr particles synthesized by using different ratios of EG and H_2O.

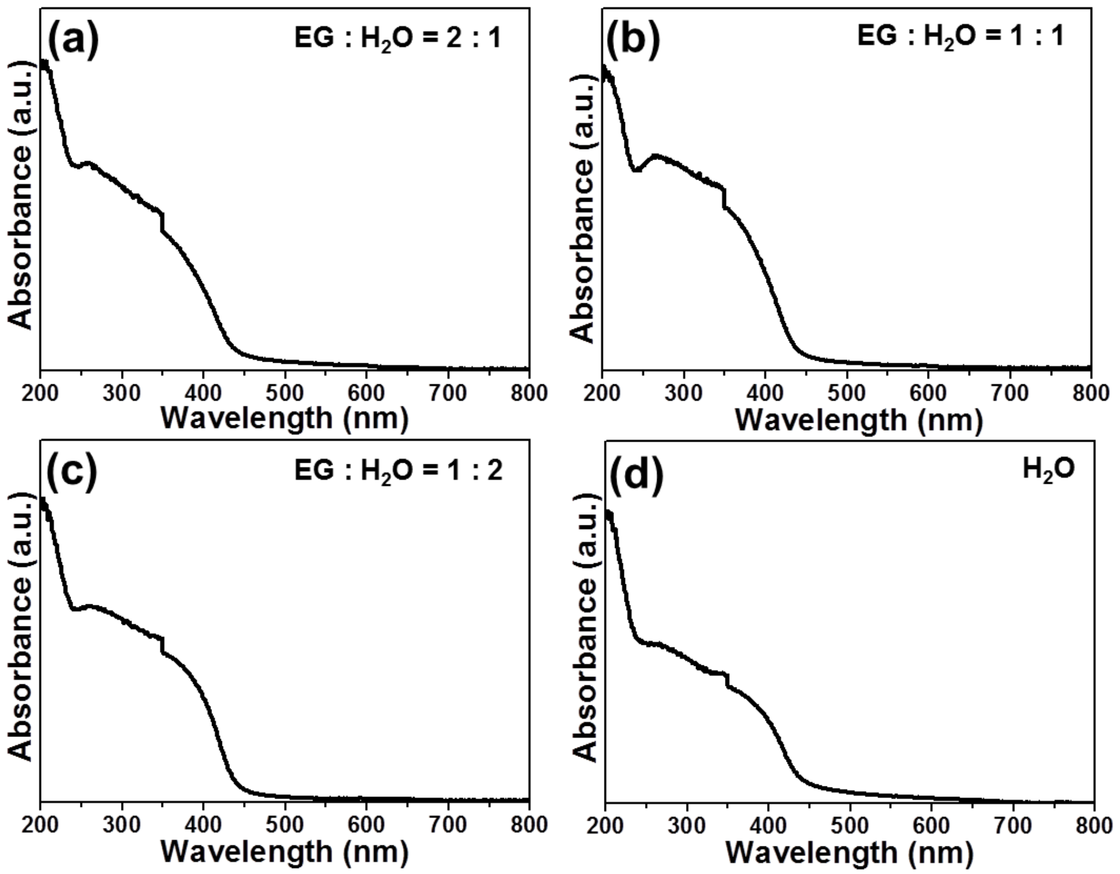

Figure S7. UV-vis absorption spectra of BiOBr compounds synthesized by using different solvents of (a) EG:H_2O = 2:1, (b) EG: H_2O = 1:1, (c) EG: H_2O = 1:2 and (d) H_2O.

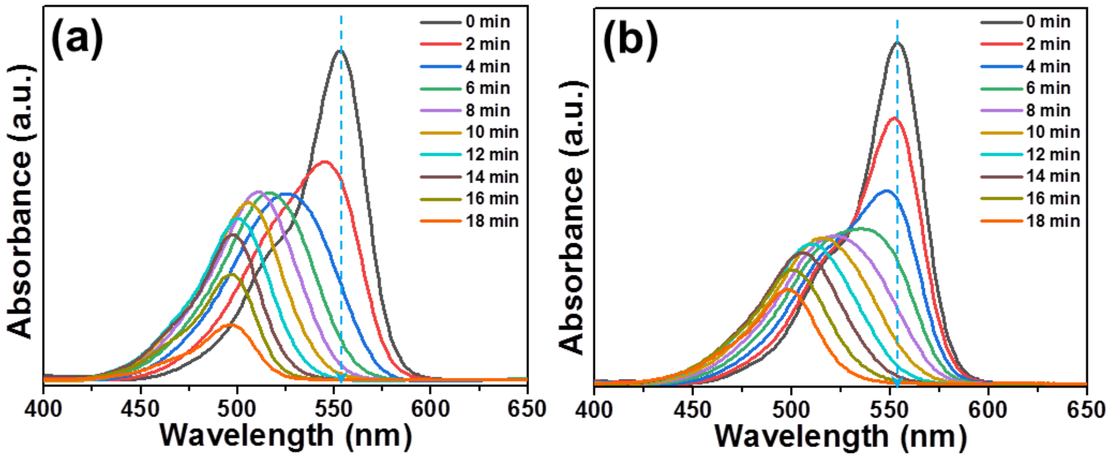

Figure S8. UV-vis absorption spectra of RhB photodegradation in the presence of BiOBr particles prepared by diverse solvents of (a) EG:H_2O = 1:1 and (b) EG:H_2O = 1:2.

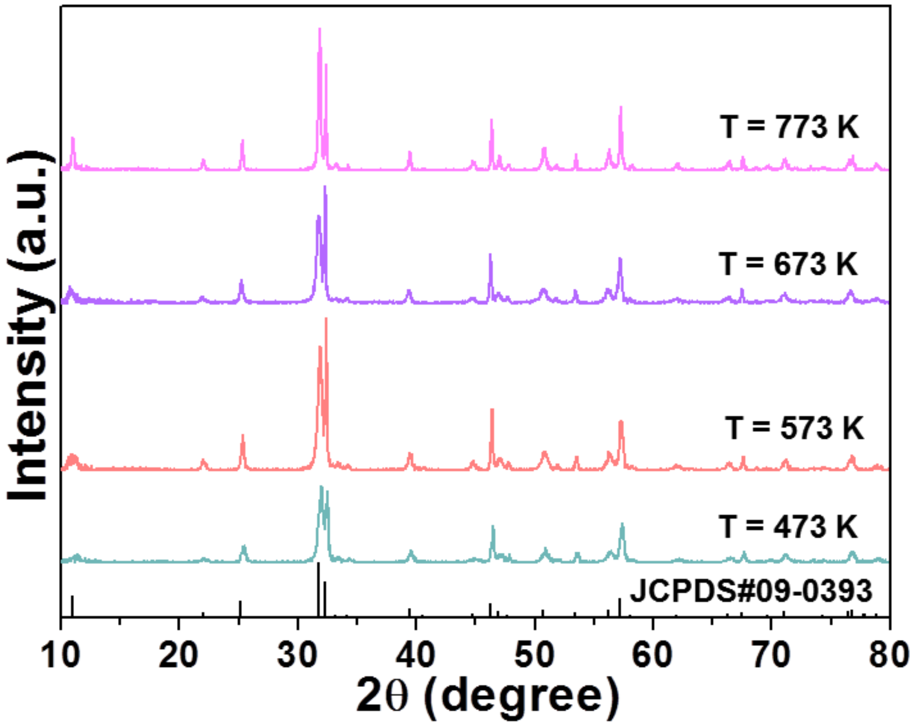

Figure S9. XRD patterns of BiOBr particles under various heat treatment temperatures.

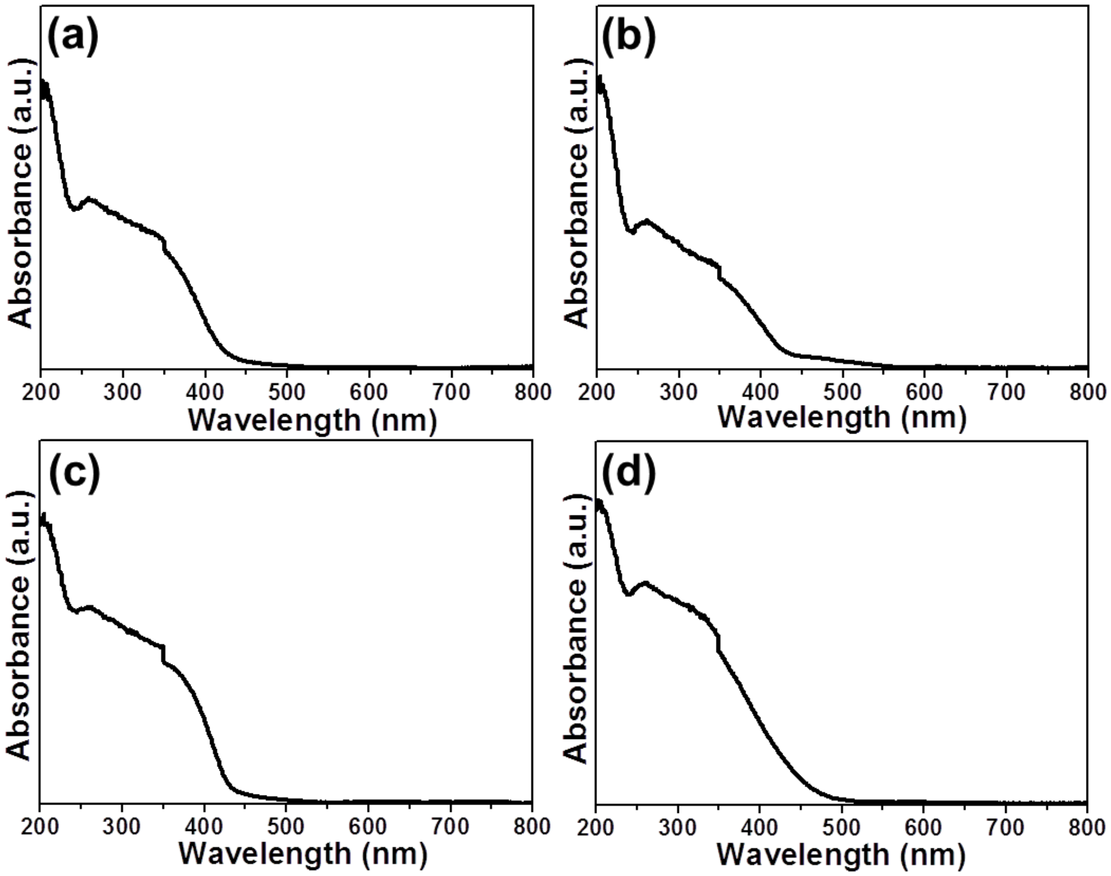

Figure S10. UV-vis absorption spectra of BiOBr particles prepared by various heat treatment temperatures of (a) T = 473 K, (b) T = 573 K, (c) T = 673 K and (d) T = 773 K.

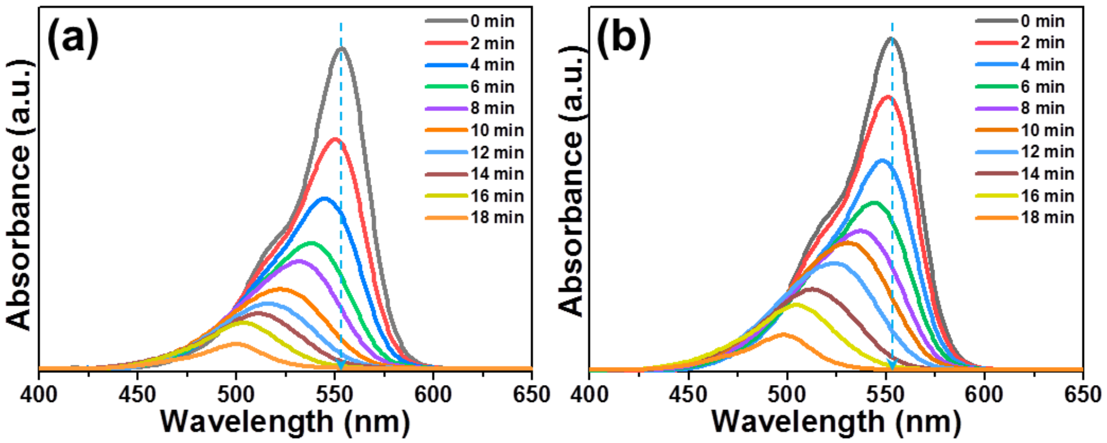

Figure S11. UV-vis absorption spectra of RhB photodegradation in the presence of BiOBr particles synthesized by diverse heating temperatures of (a) T = 573 K and (b) T = 673 K.

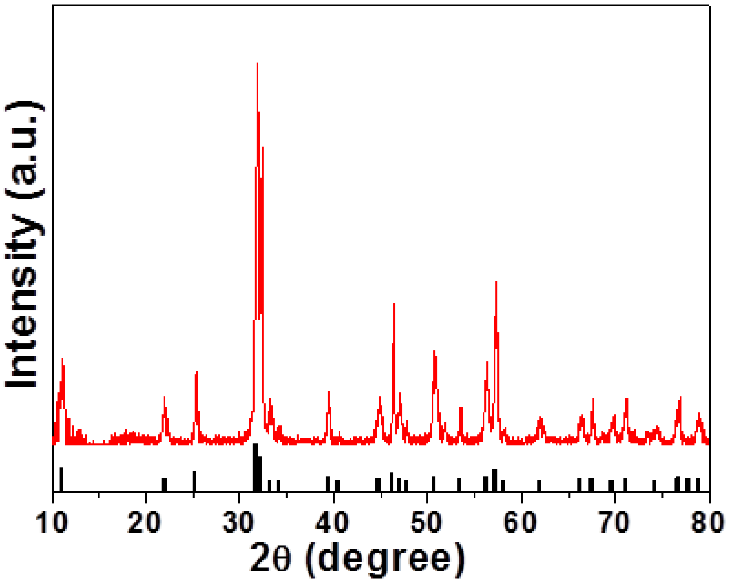

Figure S12. XRD pattern of BiOBr particles after the photodegradation process.
